# The association between mental-physical multimorbidity and disability, work productivity, and social participation in China: a panel data analysis

**DOI:** 10.1186/s12889-021-10414-7

**Published:** 2021-02-18

**Authors:** Tianxin Pan, Stewart W. Mercer, Yang Zhao, Barbara McPake, Allissa Desloge, Rifat Atun, Emily Susannah Grace Hulse, John Tayu Lee

**Affiliations:** 1 Melbourne School of Population and Global Health, The University of Melbourne, 207 Bouverie Street, Melbourne, Victoria 3010 Australia; 2 Usher Institute, College of Medicine and Veterinary Medicine, University of Edinburgh, Edinburgh, UK; 3 The George Institute for Global Health at Peking University Health Science Center, Beijing, China; 4 WHO Collaborating Centre on Implementation Research for Prevention & Control of NCDs, Melbourne, Victoria Australia; 5 Harvard T.H. Chan School of Public Health, Harvard University, Cambridge, USA; 6 Department of Primary Care and Public Health, School of Public Health, Imperial College London, London, UK

**Keywords:** Multimorbidity, Physical conditions, Mental health conditions, Productivity, Disability, Economic impact, China

## Abstract

**Background:**

The co-occurrence of mental and physical chronic conditions (mental-physical multimorbidity) is a growing and largely unaddressed challenge for health systems and wider economies in low-and middle-income countries. This study investigated the independent and combined (additive or synergistic) effects of mental and physical chronic conditions on disability, work productivity, and social participation in China.

**Methods:**

Panel data study design utilised two waves of the China Health and Retirement Longitudinal Study (2011, 2015), including 5616 participants aged ≥45 years, 12 physical chronic conditions and depression. We used a panel data approach of random-effects regression models to assess the relationships between mental-physical multimorbidity and outcomes.

**Results:**

After adjusting for socio-economic and demographic factors, an increased number of physical chronic conditions was independently associated with a higher likelihood of disability (Adjusted odds ratio (AOR) = 1.39; 95% CI: 1.33, 1.45), early retirement (AOR = 1.37 [1.26, 1.49]) and increased sick leave days (1.25 days [1.16, 1.35]). Depression was independently associated with disability (AOR = 3.78 [3.30, 4.34]), increased sick leave days (2.18 days [1.72, 2.77]) and a lower likelihood of social participation (AOR = 0.57 [0.47, 0.70]), but not with early retirement (AOR = 1.24 [0.97, 1.58]). There were small and statistically insignificant interactions between physical chronic conditions and mental health on disability, work productivity and social participation, suggesting an additive effect of mental-physical multimorbidity on productivity loss.

**Conclusion:**

Mental-physical multimorbidity poses substantial negative health and economic effects on individuals, health systems, and societies. More research that addresses the challenges of mental-physical multimorbidity is needed to inform the development of interventions that can be applied to the workplace and the wider community in China.

**Supplementary Information:**

The online version contains supplementary material available at 10.1186/s12889-021-10414-7.

## Background

Non-communicable diseases (NCDs) are the leading cause of global disease burden, contributing to approximately three-quarters of total deaths in 2017 [1], with over 85% of premature mortality occurring in low-and middle-income countries (LMICs) [2]. The prevalence of multimorbidity, defined as having two or more co-existing chronic conditions, is likely to increase given increasing exposure to risk factors (such as obesity, smoking, physical inactivity) and the ageing population in many settings [3–7]. China, the most populous country and the second-largest economy in the world, has seen rapid demographic and epidemiological transitions over the last few decades. By 2050, the projected proportion of the older population, aged 60 years and over, will dramatically increase to 35.1% from 16.2% in 2017 [8]. NCDs represent the most significant share of the burden of disease in China, comprising nearly 90% of total deaths, and 83% of total Disability-Adjusted Life Years (DALYs) lost in 2017 [9]. The prevalence of multimorbidity has been increasing rapidly in China [10–12]. A recent nationally representative Chinese study, based on 11 physical chronic conditions, found that the prevalence of multimorbidity increased from 51% in those aged 50–54 years to 71% for those aged 75 years and above [12]. Findings from the China Mental Health Survey suggested that the weighted prevalence of mental disorders among Chinese adults was 9.3% in 2013, and most mental disorders have become more common over the past 30 years [13]. The rising burden of chronic conditions and mental disorders poses a substantial threat to household welfare through increased out-of-pocket (OOP) health expenditures and reduced productivity and income. It also poses challenges to the health system in providing a combination of preventive, curative and supportive care services, as well as to the social health insurance schemes in providing financial protection for affected individuals and households. The current health system is treatment-focused and volume-driven, leading to high health expenditures [14]. China has achieved close to universal population coverage through three basic medical insurance schemes (Urban Employee Medical Insurance Scheme, Urban Residents Medical Insurance Scheme and New Rural Cooperative Medical Scheme), but the health service benefit package and patient financial reimbursements of these schemes are incomplete [15]. Patients with chronic conditions and multimorbidity are less protected and have a higher probability of incurring catastrophic health expenditures [12, 16, 17]. Also, there exist inequalities in accessing and using health care and receiving financial protection between rural and urban populations [18, 19].

Multimorbidity can involve different patterns of physical and mental chronic conditions, combinations, or clusters. Evidence from a meta-analysis suggests that the association between physical and mental chronic conditions can be bi-directional [20]. A recent study in Scotland found that among patients with multimorbidity, 4% had mental multimorbidity, while 40% had both physical and mental multimorbidity which was the most common type in those aged 55 years and less [21]. Despite the growing prevalence of mental-physical multimorbidity (i.e. the co-occurrence of mental and physical chronic conditions) in LMICs, there is little attention given to the combined impact of mental-physical multimorbidity as opposed to physical-only or mental-only chronic conditions.

The economic impact of mental-physical multimorbidity, particularly their implication on productivity is an area of emerging research inquiry. To date, the evidence on the effect of mental-physical multimorbidity is mostly from high-income countries (HICs). Evidence from HICs pointed out that multimorbidity imposes substantial economic costs, i.e. patients with multimorbidity incur large medical expenditures [22, 23] and are more likely to be absent from work [24–26]. The literature on the economic impact of mental-physical multimorbidity is relatively sparse in a LMIC setting, where the prevalence and pattern of mental-physical multimorbidity, and the ability of its health care system and social support service (such as health insurance, unemployment insurance, sick leave benefits) in mitigating the potential combined impact of physical illness and mental disorder on health and economic outcomes might be different. A recent systematic review that examined the effect of multimorbidity on productivity [27], showed that almost all previous studies were from high-income countries except one from Egypt [28]. However, the study in Egypt did not examine the combined effect of mental and physical chronic conditions on work productivity. There are no studies in China that have examined the impact of mental-physical multimorbidity on disability, work productivity, and the economy. To fill this important evidence gap, we present the first study that uses nationally-representative panel data from China, to examine (1) the independent effects of mental and physical chronic conditions on disability, work productivity, and social participation in China, and (2) the combined effects of mental-physical multimorbidity being additive or synergistic, given the independent effects of each type of chronic condition.

## Method

### Sample and data

We used data from the China Health and Retirement Longitudinal Study (CHARLS), which collects information on individuals aged 45 years or older in China and their household characteristics. The survey data included measures of physical and psychological health, demographics, socio-economic status (SES), and productivity outcomes [29]. Notably, unlike most household surveys from LMIC settings which solely rely upon self-reported data, CHARLS collects a set of biomarkers including measured blood pressure, and collects whole blood samples of all respondents who gave informed consent. At the time of our analysis, there were three waves of data (2011, 2013 and 2015) that are publicly available. However, the whole blood sample was only collected in 2011 and 2015.

We utilised two waves (2011 and 2015) of CHARLS data to construct a balanced sample. The baseline sample comprised of 17,708 individuals, of which 9971 also had data on blood pressure and the blood sample. Among then, there were 5751 individuals who remained in the 2015 follow-up wave and had their blood pressure measured and blood sample taken. After removing individuals with missing values for key independent variables on socio-demographic characteristics and weighting variables (2.3%), our final balanced sample consisted of 5616 individuals from each wave, thus a total of 11,232 pooled sample observations.

For the purpose of the study, we examined disability in an all adult sample aged 45 and older. We examined work productivity outcomes among respondents aged below 60 years, based on the mandatory retirement age for male white-collar workers in China [30]. We conducted sensitivity analyses using cut-off ages at 55 years and 65 years respectively, given that the mandatory retirement age for female white-collar workers is 55 years and the possibilities that people might continue working after the mandatory retirement age. We analysed social participation outcome among respondents who were not in employment or in the labour force (Sample flowchart is presented in Figure A1, Additional file 1).

### Variables

#### Key predictors (multimorbidity and depression)

A total of 12 physical chronic conditions were used to measure physical multimorbidity, including hypertension, diabetes and dyslipidaemia which were objectively measured based on biomarkers and blood sample information, and nine self-reported diagnosed physical chronic conditions (heart disease, stroke, cancer, chronic lung disease, digestive disease, liver disease, kidney disease, arthritis and asthma). In CHARLS, blood pressure was measured through a physical examination accompanying the survey. The systolic and diastolic blood pressure were measured and recorded three times using an electronic sphygmomanometer. For blood sample collection, three tubes of venous blood were collected from each respondent who gave consent by medically-trained staff from the Chinese Center for Disease Control and Prevention, based on a standard protocol [31]. In this study, individuals were defined as hypertensive if they had 1) a systolic blood pressure (SBP) ≥ 140 mmHg; and/or 2) a diastolic blood pressure (DBP) ≥ 90 mmHg; and/or 3) taking anti-hypertension medicines at the time of the survey [32]. Diabetes was defined by 1) a fasting plasma glucose level of ≥ 126 mg/dL (7.0 mmol/L); and/or 2) HbA1c concentration of ≥ 6.5%; and/or 3) receiving insulin treatment and/or taking medication for raised blood sugar [33, 34]. Dyslipidaemia was defined by 1) total cholesterol (TC) ≥ 240 mg/dL (6.22 mmol/L); and/or 2) low-density lipoprotein cholesterol (LDL-C) ≥ 160 mg/dL (4.14 mmol/L); and/or 3) high-density lipoprotein cholesterol (HDL-C) < 40 mg/dL (1.04 mmol/L); and/or 4) triglyceride (TG)  ≥ 200 mg/dL (2.26 mmol/L); and/or 5) taking anti-dyslipidaemia medication [35, 36]. We counted the number of physical chronic conditions reported for each participant to identify those with physical multimorbidity.

We used the presence of depression as an indicator of the presence of a mental health condition. Depression was identified using a self-reported CES-D10 score, and an individual with a score greater than ten was identified as having depression [37].

Respondents who had both depression and physical chronic conditions were defined as having mental-physical multimorbidity in this study.

#### Dependent variables

The instrumental activity of daily living (IADL) and the activity of daily living (ADL) were used to evaluate the self-reported functional disability. The abilities such as doing housework, cooking, taking medicine, shopping, and taking care of finances, which are required for living independently in the community, were used to assess the IADL. ADL refers to daily self-care tasks including eating, dressing, taking a bath, getting in and out of bed, using the toilet, and maintaining continence of urine and faeces [37]. For both the IADL and the ADL items, respondents were given a code of 1 if they reported any difficulties in any items and were identified as IADL-disability and or ADL-disability, respectively.

We examined two work productivity outcomes: (1) early retirement, which is a binary variable that indicates whether the respondent was retired at the time of the interview, and (2) the number of days missed due to sick leave at their primary job, which was calculated based on the questions “How many days of work did you miss last year due to a health problem” and the respondent’s primary work type.

Engaging in productive activities is associated with the well-being of middle-aged and older adults and important for performing valued functions to families and society [38]. Following the literature, we used an indicator of social participation to measure productive activities among older adults [39, 40]. Social participation was derived from the question “Have you done any of these activities in the last month.” A code of 1 indicates that the respondent participates in any one of the following social activities: interacting with friends; playing Mah-jong (a tile-based game developed and widely played in China), chess, or going to a community club; going to a sporting event or participating in a social group; taking part in a community-related organisation; taking part in voluntary or charity work, and attending an educational or training course.

#### Covariates

The following variables were included as covariates: year, age, gender, marital status (married and partnered, and otherwise), residency (rural or urban), Hukou (household registration system) status (agricultural Hukou or non-agricultural Hukou), geographical region (east China, middle China, west China, northeast China), family size, education (illiterate, primary school, secondary school, college and above), SES quartile (using household consumption expenditures per capita as a proxy of permanent income, Q1 being the poorest and Q4 being the richest), and work type (farming, formally employed, self-employed, family business, unemployed, retired, never worked). The included socio-demographic characteristics are those demonstrated to be associated with disability and productivity outcomes in the literature [3, 24, 27, 41].

### Statistical analyses

We summarised the mean and proportion of outcomes by the number of physical chronic conditions and the presence of depression. We used a panel data approach of random-effects logistic regression to assess the relationships between physical chronic conditions, depression and probability of reporting disability, early retirement and social participation. We applied a generalized linear model with gamma distribution and log link function to assess the relationship between physical chronic conditions, depression and the number of sick leave days, because the data on the sick leave days were highly skewed [42]. We reported the adjusted odds ratio (AOR) from logistic models and the exponentiated form of coefficients from log-gamma models with 95% confidence intervals (CI).

We fitted additional models examining whether the combined effect of both physical chronic conditions and depression was more or less than that expected (synergistic or antagonistic), given the independent burden of each (i.e. the additive effect). This was achieved by including two-way interaction terms between physical chronic conditions and depression in our regression model. A positive coefficient for the interaction terms (odds ratio > 1 in logistic regression models of disability, early retirement, and social participation and positive coefficients in the generalized linear model of sick leave days) suggests that the combined effect of two conditions is synergistic, more than the additive effect of each one of them independently. Furthermore, we tested for linearity of the number of physical chronic conditions and added a quadratic term for the number of physical chronic conditions. Predicted values of disabilities, work productivity, and social participation outcomes were estimated using coefficients from regression models. All statistical analyses were conducted using STATA 15.0. *P*-values of less than 0.05 were considered as statistically significant.

## Results

### Descriptive analysis

We analysed data from 5616 respondents. Table A1 presents the respondents’ socio-economic and demographic characteristics (see Additional file 2). The median age of the respondents was 62 years old (Interquartile range (IQR) =56–68) in 2015. There was a slightly higher percentage of female (54%) than male respondents. The majority of the respondents were married (85%) and resided in rural areas (67%). Only 29% of the respondents had attained a level of education higher than primary school, 50% of the respondents engaged in farming, and 34% of the respondents were retired.

The prevalence of multimorbidity increased from 69% in 2011 to 76% in 2015. While this may be a result of ageing (Table A1), the increase in prevalence of multimorbidity was mainly driven by presence of two or more physical chronic conditions (increased from 34 to 43%) rather than depression (34 to 33%). We also explored patterns of multimorbidity, and we found that among people with multimorbidity in 2015, 57% of them only had physical multimorbidity (increased from 50% in 2011), and 43% of them had mental-physical multimorbidity (dropped from 50% in 2015).

The unadjusted mean or proportion of disability and productivity outcomes by type of multimorbidity are reported in Table A2 (see Additional file 3). The adjusted associations are presented blow.

We tested the interactions between physical multimorbidity and mental health, however the effects were small and not statistically significant. We tested for linearity of the number of physical chronic conditions, and the quadratic term for the number of physical chronic conditions were small and not statistically significant. We therefore only present the more parsimonious model without interactions or a quadratic term as the main results (Table 1). The results from the models including interactions are presented in Table A3 (see Additional file 4).

**Table 1:** Association between multimorbidity and disability, work productivity and social participation

	Disability		Work productivity		Social participation
	Difficulties in IADLs (*n* = 11,116) AOR (95% CI)	Difficulties in ADLs (*n* = 11,162) AOR (95% CI)	Early retirement (*n* = 5261) AOR (95% CI)	Number of days of sick leave (*n* = 4141) mean (95% CI)	Social participation (*n* = 3301) AOR (95% CI)
Number of physical NCDs	**1.27 (1.22, 1.32)**	**1.39 (1.33, 1.45)**	**1.37 (1.26, 1.49)**	**1.25 (1.16, 1.35)**	**1.06 (1.00, 1.13)**
Depression	**3.59 (3.16, 4.07)**	**3.78 (3.30, 4.34)**	1.24 (0.97, 1.58)	**2.18 (1.72, 2.77)**	**0.57 (0.47, 0.70)**
2015	**1.21 (1.08, 1.35)**	**1.46 (1.29, 1.66)**	**1.58 (1.28, 1.96)**	1.07 (0.85, 1.35)	0.98 (0.82, 1.17)
Age group (ref: 45–54)					
Age 55–64	**1.43 (1.21, 1.69)**	**1.67 (1.39, 2.02)**	**1.96 (1.54, 2.49)**	0.92 (0.72, 1.17)	0.92 (0.68, 1.24)
Age 65–74	**2.21 (1.82, 2.67)**	**2.49 (2.02, 3.08)**			0.94 (0.68, 1.29)
Age 75+	**4.08 (3.14, 5.30)**	**3.85 (2.89, 5.12)**			**0.66 (0.45, 0.97)**
Female gender	**1.46 (1.27, 1.67)**	**1.19 (1.02, 1.38)**	**4.60 (3.39, 6.26)**	0.89 (0.70, 1.13)	1.06 (0.85, 1.32)
Married	1.03 (0.86, 1.23)	0.97 (0.80, 1.18)	0.97 (0.59, 1.58)	1.36 (0.85, 2.19)	**0.74 (0.57, 0.95)**
Agricultural hukou	1.09 (0.91, 1.32)	1.18 (0.97, 1.44)	**0.32 (0.23, 0.45)**	1.33 (0.92, 1.92)	**0.48 (0.37, 0.62)**
Rural residency	**1.41 (1.21, 1.63)**	**1.23 (1.04, 1.44)**	**0.24 (0.18, 0.33)**	1.25 (0.97, 1.63)	1.19 (0.95, 1.50)
Region (ref:east China)					
Middle China	**1.20 (1.02, 1.42)**	**1.88 (1.57, 2.25)**	0.81 (0.59, 1.12)	1.11 (0.84, 1.48)	0.93 (0.73, 1.20)
West China	1.12 (0.95, 1.32)	1.14 (0.95, 1.37)	**0.58 (0.42, 0.81)**	1.17 (0.89, 1.55)	**0.66 (0.51, 0.86)**
Northeast China	**1.78 (1.37, 2.31)**	**1.73 (1.30, 2.32)**	1.11 (0.66, 1.86)	1.35 (0.82, 2.24)	0.76 (0.53, 1.10)
Family size (ref: 1, 2 members)					
3–4 members	1.07 (0.93, 1.23)	1.06 (0.91, 1.23)	1.01 (0.77, 1.33)	0.90 (0.69, 1.17)	0.95 (0.77, 1.18)
4+ members	**1.22 (1.04, 1.44)**	0.95 (0.79, 1.14)	**1.51 (1.09, 2.10)**	1.06 (0.78, 1.46)	1.08 (0.83, 1.40)
Education level (ref:illiterate)					
Primary	**0.60 (0.51, 0.71)**	**0.75 (0.63, 0.90)**	0.83 (0.58, 1.19)	0.79 (0.58, 1.07)	1.27 (0.98, 1.65)
Secondary	**0.48 (0.39, 0.58)**	**0.61 (0.49, 0.75)**	1.20 (0.86, 1.69)	0.79 (0.59, 1.08)	**1.40 (1.04, 1.87)**
Tertiary	**0.38 (0.28, 0.52)**	**0.43 (0.31, 0.60)**	0.88 (0.56, 1.36)	0.76 (0.52, 1.12)	**2.79 (1.84, 4.25)**
HH consumption per capita (ref:Q1)					
Q2	1.05 (0.89, 1.22)	0.99 (0.84, 1.18)	0.84 (0.60, 1.18)	1.13 (0.83, 1.55)	1.18 (0.90, 1.54)
Q3	1.06 (0.90, 1.25)	0.89 (0.75, 1.06)	1.10 (0.79, 1.53)	1.03 (0.76, 1.40)	1.09 (0.83, 1.42)
Q4 (richest)	1.11 (0.94, 1.32)	1.00 (0.83, 1.20)	**1.68 (1.21, 2.34)**	**1.49 (1.07, 2.07)**	1.30 (0.99, 1.70)
Work type (ref:farming)					
Formally Employed				**0.32 (0.23, 0.44)**	
Self-employed				**0.65 (0.43, 0.99)**	
Family business				1.30 (0.63, 2.70)	

The regression model is adjusted for all socio-demographic covariates. Bold font indicate significance at 5% level

Generalized linear model with gamma distribution and log link function is used to estimate the association between multimorbidity and the number of days of sick leave at main job. Random-effect logistic models are used for other outcomes

*AOR* Adjusted odds ratio, *CI* Confidence interval

### Mental-physical multimorbidity and disability

Table 1 shows the adjusted odd ratios for the associations between mental-physical multimorbidity and disability. An increasing number of physical chronic conditions (AOR = 1.27, 95% CI: 1.22, 1.32), and the presence of depression (AOR = 3.59, 95% CI: 3.16, 4.07) was associated with a higher risk of IADL disability. Similarly, an increasing number of physical chronic conditions (AOR = 1.39, 95% CI: 1.33, 1.45), and the presence of depression (AOR = 3.78, 95% CI: 3.30, 4.34) was also associated with a higher risk of ADL disability.

**Fig. 1:**
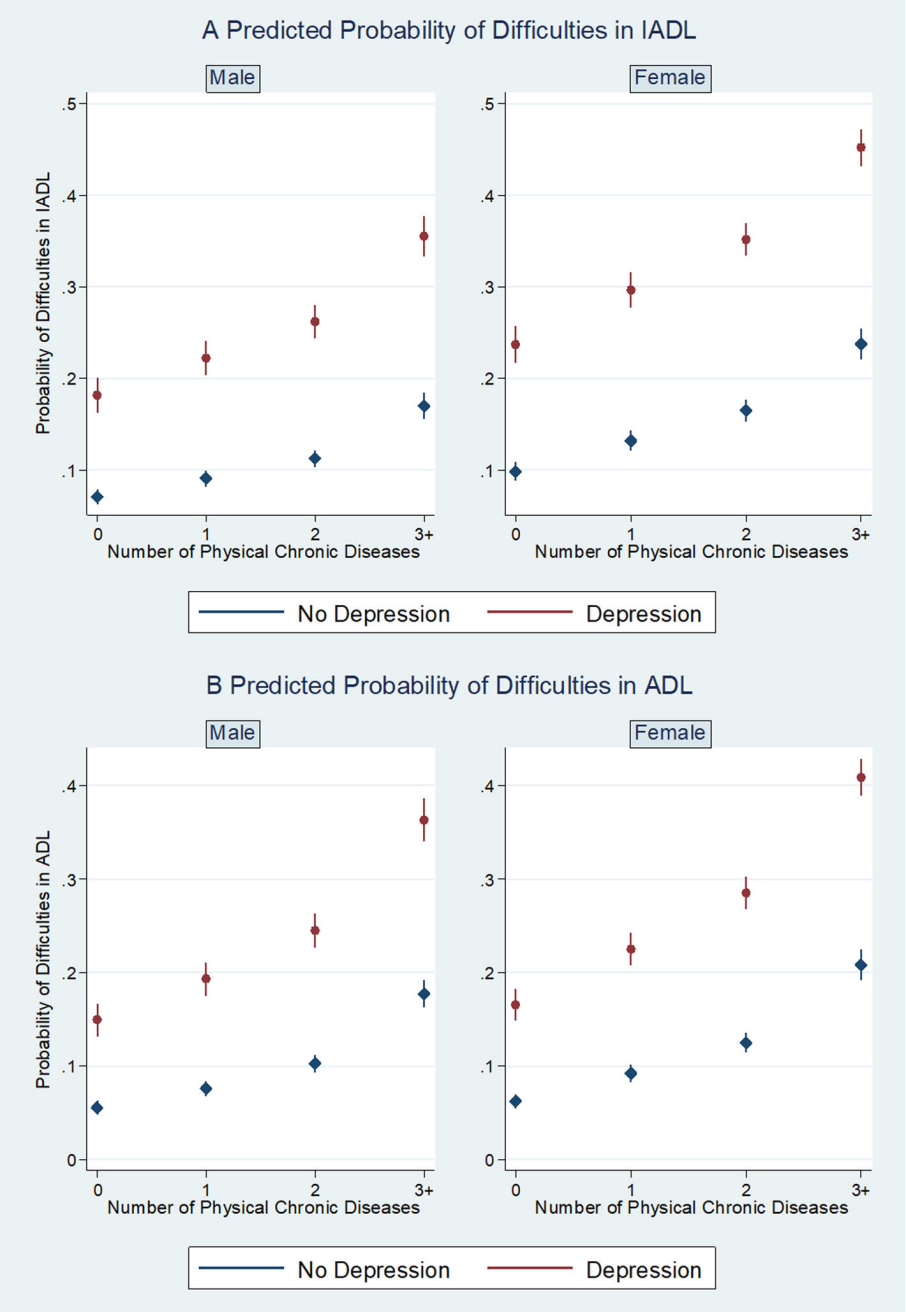
The associations between mental-physical multimorbidity and disability, by gender. **a** Predicted probability of difficulties in instrumental activity of daily living (IADL) by the number of physical chronic conditions and depression, stratified by gender group, China Health and Retirement Longitudinal Study, 2011 and 2015. Random-effect logistic models are used. Adjusted for all socio-demographic covariates. **b** Predicted probability of difficulties in activity of daily living (ADL) by the number of physical chronic conditions and depression, stratified by gender group, China Health and Retirement Longitudinal Study, 2011 and 2015. Random-effect logistic models are used. Adjusted for all socio-demographic covariates

The predicted probability of reporting limitations in IADLs increased from 0.07 for males and from 0.10 for females without any physical chronic conditions and depression to 0.36 for males and 0.45 for females with more than two physical chronic conditions and depression respectively (Fig. 1a). Similarly, compared to those without physical chronic conditions and depression, the predicted probability of reporting limitations in ADLs was much higher for individuals with more than two physical chronic conditions and with depression (Fig. 1b).

### Mental-physical multimorbidity and work productivity

Table 1 also shows that among respondents aged under 60 years, early retirement was associated with an increasing number of physical chronic conditions (AOR = 1.37, 95% CI: 1.26, 1.49), but not associated with depression (AOR = 1.24, 95% CI: 0.97, 1.58). The odds ratio of reporting early retirement was nearly five times higher for females compared to males (AOR = 4.60, 95% CI: 3.39, 6.26). We found that an increasing number of physical chronic conditions was independently associated with an increased number of sick leave days (mean number of days = 1.25, 95% CI: 1.16, 1.35). The presence of depression was also associated with an increased number of sick leave days (mean number of days = 2.18, 95% CI: 1.72, 2.77). Depression was associated with a higher number of sick leave days than one physical chronic condition. In addition, respondents who engaged in the formal sectors or were self-employed were associated with fewer sick leave days compared to those engaged in farming. When we using a sample of respondents under 55 years and using a sample of respondents under 65 years, the results were fairly similar to our main results (see Table A4 in Additional file 5).

**Fig. 2:**
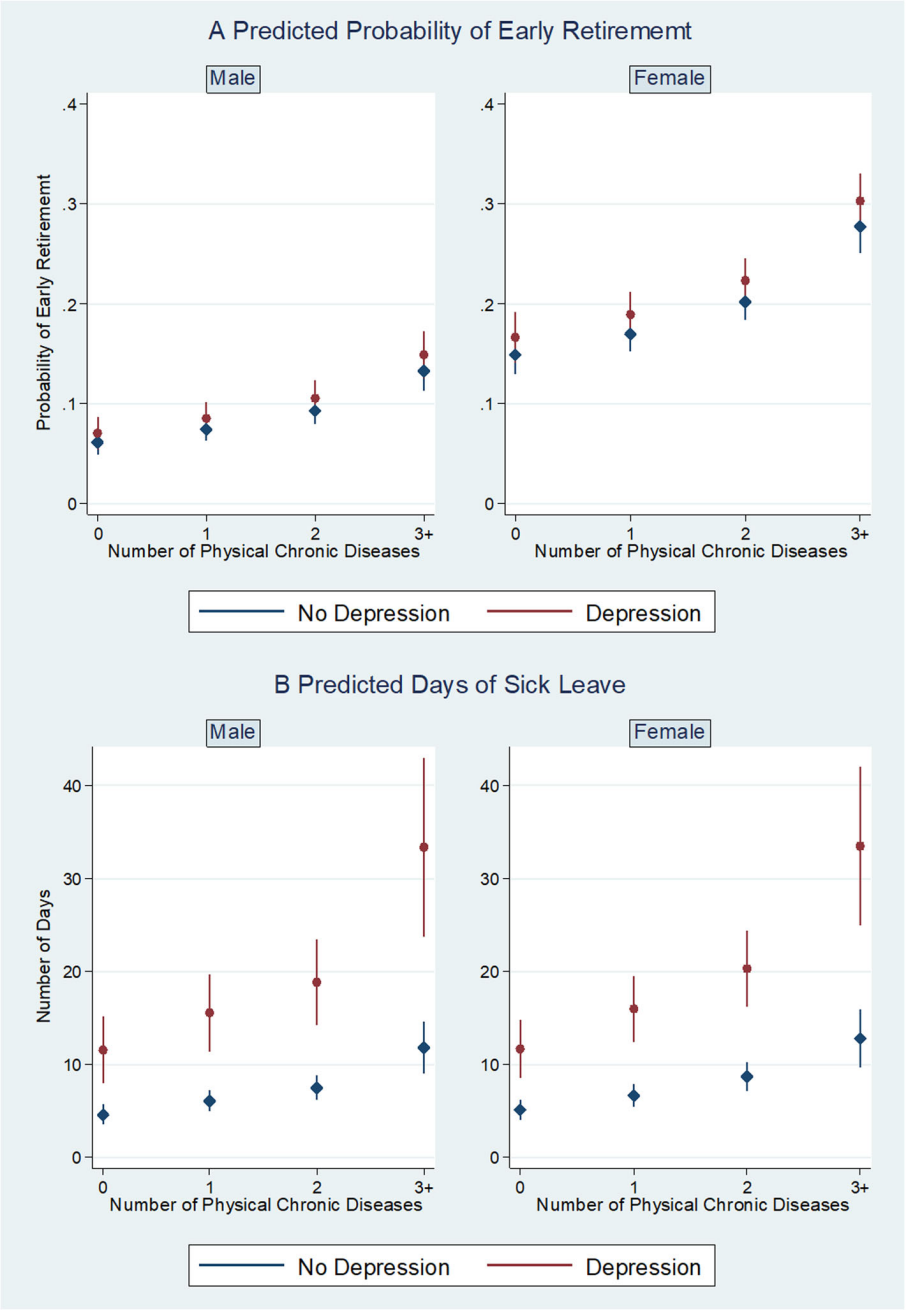
The associations between mental-physical multimorbidity and work productivity, by gender. **a** Predicted probability of early retirement by the number of physical chronic conditions and depression, stratified by gender group, China Health and Retirement Longitudinal Study, 2011 and 2015. Random-effect logistic model is used. Adjusted for all socio-demographic covariates. **b** Predicted number of sick leave days by the number of physical chronic conditions and depression, stratified by gender group, China Health and Retirement Longitudinal Study, 2011 and 2015. Generalized linear model with gamma distribution and log link function is used. Adjusted for all socio-demographic covariates

Among the working-age population, the predicted probability of reporting early retirement increased from 0.06 for males and from 0.15 for females without any physical chronic conditions and depression to 0.15 for males and 0.30 for females with more than two physical chronic conditions and depression respectively (Fig. 2a). Similarly, compared to those without physical chronic conditions and depression, female respondents who had depression and had more than two physical chronic conditions had more extended sick leave (33.51 days vs 4.62, Fig. 2b), compared to males without physical chronic conditions and depression.

### Mental-physical multimorbidity and social participation

**Fig. 3:**
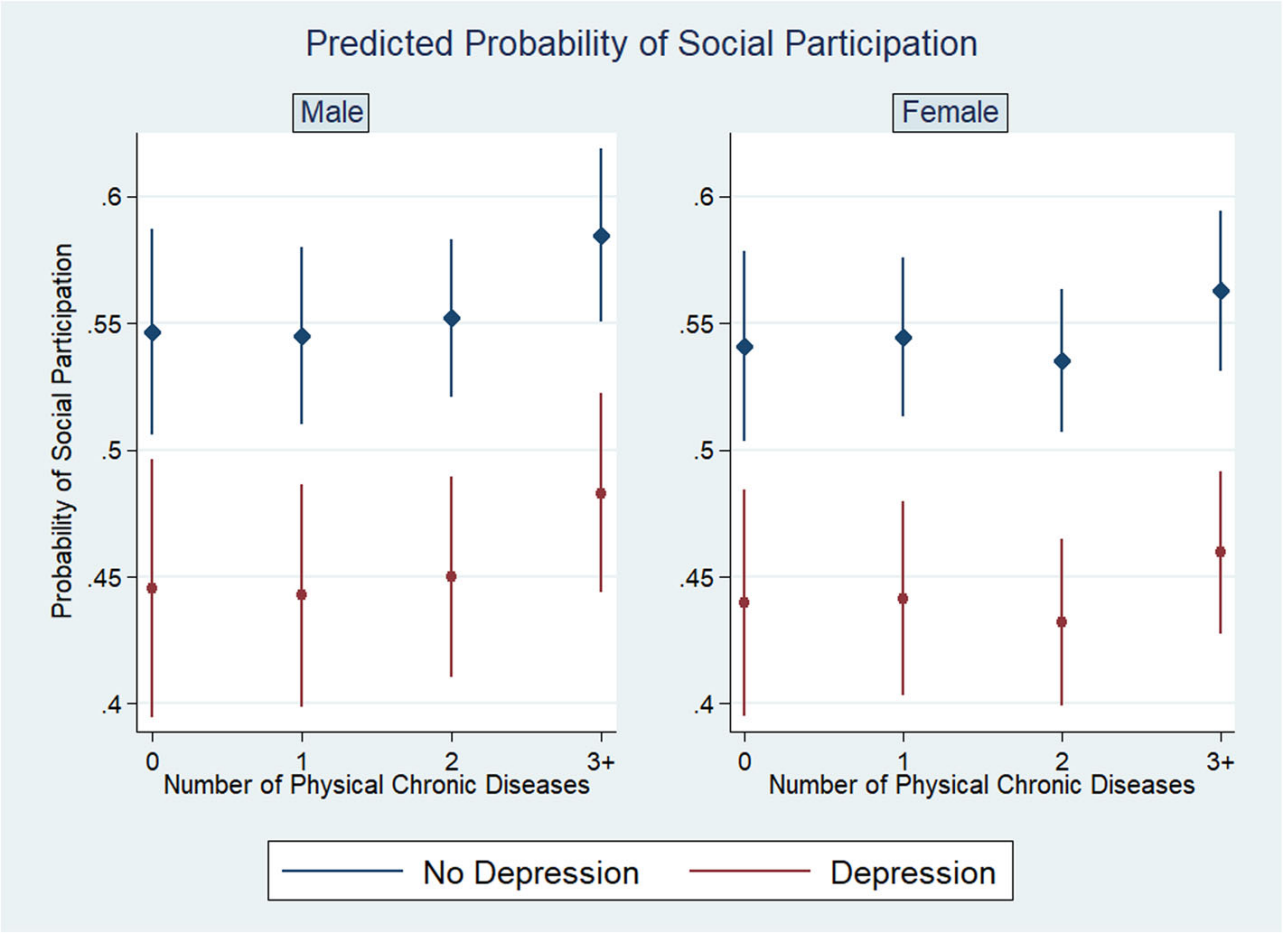
The associations between mental-physical multimorbidity and social participation, by gender. Predicted probability of social participation by the number of physical chronic conditions and depression, stratified by gender group, China Health and Retirement Longitudinal Study, 2011 and 2015. Random-effect logistic models are used. Adjusted for all socio-demographic covariates

We found that the presence of depression was associated with a substantial decrease in the likelihood of social participation in those who were not working (AOR = 0.57, 95% CI: 0.47, 0.70). We also found a small but statistically significant association between an increasing number of physical chronic conditions and likelihood of social participation (AOR = 1.06, 95% CI: 1.00, 1.13). As shown in Fig. 3, the predicted probability of social participation was 0.55 for males (0.54 for females) without physical chronic conditions and without depression, and dropped to 0.48 for males (0.46 for females) with more than two physical conditions and depression.

## Discussion

This study found that the prevalence of multimorbidity was 76% among Chinese aged over 45 years in 2015. Among them, 57% had two or more physical chronic conditions, and 43% had both mental and physical chronic conditions. Physical chronic conditions were independently associated with a higher likelihood of disability, early retirement, increased sick leave days and participation in social activity. The presence of depression was also independently associated with disability, increased sick leave days and a lower likelihood of social participation. Mental-physical multimorbidity showed additive effects on disability, work loss and social participation.

We found the overall prevalence of multimorbidity in 2015 to be 76%, which was higher than the results from other studies using either the same or similar dataset [11, 12, 43, 44]. For example, Zhao et al. used the same dataset and found the prevalence of multimorbidity, physical multimorbidity and mental-physical multimorbidity in 2015 to be 62, 39 and 32% respectively [12, 43]. The main reason is that these studies used self-reported measures of physical chronic conditions, whereas our study used objective measures of diabetes and dyslipidaemia based on a whole blood sample. Studies that used nationally-representative sample of screening or surveillance data have shown that the prevalence of hypertension, diabetes and dyslipidaemia were high among Chinese adults (45, 12 and 43% respectively), but awareness rates were very low, with only one-third of patients being aware of their conditions [34, 45–47].

Our findings are generally in line with those of a small number of previous studies from HICs, which showed that physical chronic conditions and depression were independently associated with the number of sick leave days, and the effect of depression on work productivity loss was larger compared to an increased number of physical chronic conditions [24, 41]. The evidence on the combined effect of mental-physical multimorbidity on work productivity loss (including absenteeism and/or presenteeism) is mixed from HICs and varies across the type of physical chronic conditions, productivity outcome considered and study sample. Several studies reported that mental-physical multimorbidity led to a mainly additive increase in work loss. For example, Demyttenaere et al. used a general population sample from six European countries and found that respondents with depression only and those with painful physical symptoms only reported 4.5 and 3.6 work-loss days respectively, whereas respondents with both conditions reported 9.4 work-loss days per month [24]. Buist-Bouwman et al. used a general population sample aged between 18 and 64 from the Netherlands and examined the independent and combined effects of different physical and mental conditions on work loss. They found that mental disorders led to significantly more work-loss days than hypertension only (28.3 days vs 5.6 days) and asthma only (18.6 days vs 5.4 days), and was comparable with diseases of the digestive system only (14.2 days vs 14.1 days) in the past 12 months. The effect of mental-physical multimorbidity was additive for asthma (18.6 days) and digestive system diseases (33.3 days) but synergistic for hypertension (28.3 days). However, an Australian study using a sample of the working population and WHO Health and Productivity Questionnaire found synergistic effects on absenteeism for some physical conditions (such as cancer, high cholesterol) when co-morbid with psychological distress. They also found synergistic and larger effects on presenteeism than absenteeism for every physical conditions included in their study when co-morbid with psychological distress [25].

To date, there have been no studies exploring the combined effect of mental-physical multimorbidity on productivity in China or other LMICs. We add to the existing literature that the combined effects are mainly additive in China. One distinction between LMICs and HICs is that a higher number of people engaged in the informal sector such as farming in LMICs [48, 49]. Our analysis showed that compared to people who work in the formal sector, those engaged in farming were more likely to have an increased number of sick leave days. However, there existed the possibility that employees in the formal sectors may show up for work while sick, which can lead to presenteeism [50]. Furthermore, we found that respondents with physical chronic conditions were more likely to retire early, and the effects were substantially larger on females compared to males. This is in line with studies from HICs, which showed similar findings that females with multimorbidity were more likely to transition out of a full-time job [51].

Consistent with earlier studies examining the effect of multimorbidity on disability in both LMICs and HICs settings [3, 52–54], our study found that both physical multimorbidity and depression was positively associated with disability among Chinese mid-aged and elderly population. We found that the combined effect of the co-existence of mental and physical chronic conditions on IADL and ADL was additive of each independently. However, evidence on the combined effects on disability is rather mixed, with additive effects [3, 55, 56] and synergistic effects [53, 57] both being found in literature. These studies differ in the type of physical chronic conditions included and disability measure used.

Our findings on the negative association between depression and social participation align with other studies from HICs [58]. However, we also found a small but positive association between an increasing number of physical chronic conditions and social participation, whereas most existing studies showed physical chronic conditions to be associated with lower participation in social activities. One explanation might be attributed to the types of activities which were considered as social participation in the CHARLS survey, which varied widely in terms of the physical ability needed. An alternative explanation is that there are other important determinants, such as education that may have a greater impact on social participation [58].

Recent literature reviews has pointed out that the literature on multimorbidity and work productivity loss is relatively sparse in LMICs [23, 27]. To the best of our knowledge, this is the first study that examined the impact of mental-physical multimorbidity across disability, work productivity, and social participation outcomes using a robust panel study design in China. However, the study has several caveats. First relates to the use of retrospective self-reported data and potential measurement error. We used a self-reported number of sick leave days which may open to recall bias, particularly among those working in the informal sector. We used objective measures of hypertension, diabetes and dyslipidaemia; however other physical chronic conditions still relied on self-reported data which may lead to underestimation particularly among those from lower SES and educational backgrounds and rural areas due to restricted access to health care services [17]. Future work that links household survey data with surveillance systems or hospital administrative data could provide high-quality information on patient health and the character of the disease itself, thus help to address the measurement error associated with self-reported data. Second, reverse causality may exist in the relationship between mental-physical multimorbidity on work productivity and social participation. For instance, early retirement has impacts on mental health. People move to retirement early show improved subjective health status and mental health [59, 60], on the other hand, people who experience reduced work hours and job loss tend to have poor mental health [59, 61]. Third, the presence of unobserved factors of multimorbidity and work and social productivity measures, such as personality, can bias our estimation. The availability of panel data can normally help attenuate the effects of unobserved heterogeneity at the individual level by applying a fixed-effects model. However, by applying fixed-effects model we cannot identify the impact of factors of interest that lack variation over time, such as gender and education. We acknowledge that our results remain subject to bias in terms of measurement error and omitted variables, and our study does not infer causation and further studies are needed to look at the causal effect of multimorbidity on productivity loss. Future research would benefit from more extended panels, which enables observations of the onset of mental-physical multimorbidity and its long-term impact on productivity loss, and vice versa. Fourth, we have used a count of physical chronic conditions as a measure of physical multimorbidity, which did not account for different types and the levels of severity of the chronic conditions. In addition, CHARLS does not contain other measures on mental health conditions except for depression, so our estimated effects of having depression may also capture a wider effect of mental health conditions. The literature shows that the combined effect of mental-physical multimorbidity on disability and work productivity differ across types of conditions [24, 41, 53]. Future research that considers different combinations of chronic conditions and include more mental conditions can enrich our understanding of the economic impact of mental-physical multimorbidity. Lastly, our estimation on the association between multimorbidity and work productivity was among respondents aged between 45 and 60 years. Due to the fact that CHALRS only focused on people aged 45 years and above, we were not able to estimate the effects of multimorbidity on work productivity on a younger population, where the magnitude of the effects might be different [62, 63]. There is a need for further research on the economic impact in younger population, particularly from LMICs [4].

Our findings have important implications for public health policy and research. The findings that more than 40% of respondents with multimorbidity had both mental and physical chronic conditions in China highlight the importance to address the co-existing mental-physical multimorbidity. The healthcare system in China is more hospital-centric and with a single disease model, rather than a strong primary healthcare system led by generalist physicians working with multi-disciplinary teams. Efficient management of mental-physical multimorbidity needs an integrated care model and implement interventions in different settings, including clinical settings, community and workplace. Protecting the physical health of people with mental illness and protecting the mental health of people with physical chronic conditions should be considered a priority to reduce the economic impact of mental-physical multimorbidity to individuals, employers and the whole society, particularly in low-income and middle-income settings. Public health strategies aimed at addressing mental-physical multimorbidity in the workplace should be seen as an ‘investment’ rather than a cost, with the costs of the intervention likely to be ‘offset’ by long-term cost savings achieved from improved health and productivity [64]. In China, these interventions may include supporting a healthy work environment, promoting workplace physical activity, and providing on-site counselling services as well as resilience and coping skills training [65]. More research that addresses the challenges of mental-physical multimorbidity is needed to inform the development of interventions that can be applied to the workplace and the wider community in China, and effectiveness trials in workplace samples are needed to evaluate the cost-effectiveness of interventions to screen and manage mental-physical multimorbidity among employees.

## Conclusion

Mental-physical multimorbidity poses substantial negative health and economic effects on individuals, health systems, and societies. More research that addresses the challenges of mental-physical multimorbidity is needed to inform the development of interventions that can be applied to the workplace and the wider community in China.

## Supplementary Information

**Additional file 1: Figure A1.** Sample Flowchart. Figure A1 presents the sample flowchart, and the number of observations for each outcome in this study.

**Additional file 2.** Statistic summary of sample characteristics. Table A1 presents the sample characteristics of the analytical sample in this study.

**Additional file 3.** Statistic summary of disability, work productivity and social participation by physical and mental conditions and gender. Table A2 presents the unadjusted mean or proportion of disability and productivity outcomes by type of multimorbidity.

**Additional file 4.** Association between multimorbidity and disability, work productivity and social participation, models including interactions between physical conditions and depression. Table A3 presents the results from the model that included interactions between number of physical chronic conditions and depression.

**Additional file 5.** Sensitivity analyses on the association between multimorbidity and productivity loss among different age groups. Table A4 presents the results of the sensitivity analyses on the association between multimorbidity and productivity loss using different samples. We repeated the analysis using a sample of respondents aged under 55 years, which is the mandatory retirement age for female white-collar workers. In addition, we used a cut-off at 65 years, given the possibilities that people may continue working after the mandatory retirement age.

## Data Availability

The data that support the findings of this study are available in the [http://charls.pku.edu.cn/index/en.html]. [The CHARLS] Yaohui Zhao, et al.; 2018; Harmonized CHARLS; the Gateway to Global Aging Data; Version C; http://charls.pku.edu.cn/pages/data/harmonized_charls/en.html.

## Abbreviations

ADL: Activity of daily living
AOR: Adjusted odds ratio
CHARLS: China Health and Retirement Longitudinal Study
CI: Confidence intervals
HICs: High-income countries
IADL: Instrumental activity of daily living
LMICs: Low-and middle-income countries
NCDs: Non-communicable diseases
