# Supplementary material for: The association between mental-physical multimorbidity and disability, work productivity, and social participation in China: a panel data analysis
Source: BMC Public Health. 2021 Feb 18;21:376. doi: 10.1186/s12889-021-10414-7 (PMC7890601; doi:10.1186/s12889-021-10414-7)
Supplement: Supplementary file 1 — Additional file 1: Figure A1. Sample Flowchart. Figure A1 presents the sample flowchart, and the number of observations for each outcome in this study. [file 12889_2021_10414_MOESM1_ESM.docx]

Additional File 1:

Title: Figure A1 Sample Flowchart

IADL

(n=11116)

Disability

Sample: aged 45 and older

(n=11232)

Early retirement

(n=5261)

Work Productivity

Sample: aged below 60 years

(n=5272 )

Sick leave days

(n=4141)

ADL

(n=11162)

Social participation

(n=3301)

Social Participation

Sample: not in work force (n=3401)

In the pooled sample of 2011 & 2015

(n=11232)

CHARLS 2011

CHARLS 2015

Respondents who answered the questionnaire (N = 17708)

Respondents who answered the questionnaire (N = 21100)

Respondents who had blood pressure measured and blood sample taken (N = 9971)

Respondents who had blood pressure measured and blood sample taken (N =13096 )

Respondents who remained in both wave and with blood pressure measured and blood sample taken

(N = 5751)

Respondents who remained in both wave and with complete information on covariates (After imputation)

(N = 5616)

N: Number of respondents; n: Number of observations

CHARLS: China Health and Retirement Longitudinal Study

**Figure A1 Sample Flowchart**
