# Supplementary material for: The association between mental-physical multimorbidity and disability, work productivity, and social participation in China: a panel data analysis
Source: BMC Public Health. 2021 Feb 18;21:376. doi: 10.1186/s12889-021-10414-7 (PMC7890601; doi:10.1186/s12889-021-10414-7)
Supplement: Supplementary file 2 — Additional file 2. Statistic summary of sample characteristics. Table A1 presents the sample characteristics of the analytical sample in this study. [file 12889_2021_10414_MOESM2_ESM.docx]

Additional File 2:

Title: Statistic summary of sample characteristics a pooled sample of year 2011 and 2015

| Table A1 Sample characteristics of a pooled sample of year 2011 and 2015 | | | |
| --- | --- | --- | --- |
|  | **Full sample (N=11232)** | **2011 (N=5616)** | **2015 (N=5616)** |
| Multimorbidity | 8131 (72%) | 3865 (69%) | 4266 (76%) |
| Physical only multimorbidity | 4371 (39%) | 1935 (34%) | 2436 (43%) |
| Mental-physical multimorbidity | 3760 (33%) | 1930 (34%) | 1830 (33%) |
| Depression | 4083 (36%) | 2136 (38%) | 1947 (35%) |
| Age group |  |  |  |
| Age 45-54 | 3049 (27%) | 1897 (34%) | 1152 (21%) |
| Age 55-64 | 4593 (41%) | 2340 (42%) | 2253 (40%) |
| Age 65-74 | 2713 (24%) | 1091 (19%) | 1622 (29%) |
| Age 75+ | 877 (8%) | 288 (5%) | 589 (10%) |
| Sex |  |  |  |
| Male | 5182 (46%) | 2591 (46%) | 2591 (46%) |
| Female | 6050 (54%) | 3025 (54%) | 3025 (54%) |
| Marital status |  |  |  |
| Married | 9773 (87%) | 4982 (89%) | 4791 (85%) |
| Single, divorced, widow | 1459 (13%) | 634 (11%) | 825 (15%) |
| Residency |  |  |  |
| Urban | 3732 (33%) | 1866 (33%) | 1866 (33%) |
| Rural | 7500 (67%) | 3750 (67%) | 3750 (67%) |
| Hukou |  |  |  |
| Non-agricultural | 2101 (19%) | 868 (15%) | 1233 (22%) |
| Agricultural | 9131 (81%) | 4748 (85%) | 4383 (78%) |
| Region |  |  |  |
| East China | 3280 (29%) | 1640 (29%) | 1640 (29%) |
| Middle China | 3512 (31%) | 1756 (31%) | 1756 (31%) |
| West China | 3648 (32%) | 1824 (32%) | 1824 (32%) |
| Northeast China | 792 (7%) | 396 (7%) | 396 (7%) |
| Family size |  |  |  |
| 1-2 members | 4572 (41%) | 2095 (37%) | 2477 (44%) |
| 3-4 members | 4265 (38%) | 1807 (32%) | 2458 (44%) |
| 4+ members | 2395 (21%) | 1714 (31%) | 681 (12%) |
| Educational level |  |  |  |
| Illiterate | 5386 (48%) | 2693 (48%) | 2693 (48%) |
| Primary | 2622 (23%) | 1311 (23%) | 1311 (23%) |
| Secondary | 2236 (20%) | 1118 (20%) | 1118 (20%) |
| Tertiary | 988 (9%) | 494 (9%) | 494 (9%) |
| Household consumption quantile |  |  |  |
| Q1 (poorest) | 2806 (25%) | 1404 (25%) | 1402 (25%) |
| Q2 | 2806 (25%) | 1405 (25%) | 1401 (25%) |
| Q3 | 2803 (25%) | 1403 (25%) | 1400 (25%) |
| Q4 (richest) | 2804 (25%) | 1404 (25%) | 1400 (25%) |
| Work Type |  |  |  |
| Farming | 5947 (53%) | 3184 (57%) | 2763 (50%) |
| Formally Employed | 1126 (10%) | 561 (10%) | 565 (10%) |
| Self-employed | 523 (5%) | 304 (5%) | 219 (4%) |
| Family business | 210 (2%) | 78 (1%) | 132 (2%) |
| Unemployed | 22 (0%) | 19 (0%) | 3 (0%) |
| Retired | 3242 (29%) | 1337 (24%) | 1905 (34%) |
| Never work | 137 (1%) | 133 (2%) | 4 (0%) |
| Note: Data are n (%) |  |  |  |
