# Supplementary material for: The association between mental-physical multimorbidity and disability, work productivity, and social participation in China: a panel data analysis
Source: BMC Public Health. 2021 Feb 18;21:376. doi: 10.1186/s12889-021-10414-7 (PMC7890601; doi:10.1186/s12889-021-10414-7)
Supplement: Supplementary file 3 — Additional file 3. Statistic summary of disability, work productivity and social participation by physical and mental conditions and gender. Table A2 presents the unadjusted mean or proportion of disability and productivity outcomes by type of multimorbidity. [file 12889_2021_10414_MOESM3_ESM.docx]

Additional File 3

Title: Statistic summary of disability, work productivity and social participation by physical and mental conditions and gender

| Table A2 Statistic summary of disability, work productivity and social participation by physical and mental conditions | | | | | |
| --- | --- | --- | --- | --- | --- |
|  | **No physical NCDs  no depression (N=962)** | **Depression (N=4083)** | **Multimorbidity (N=8131)** | **Physical-only multimorbidity (N=4371)** | **Mental-physical multimorbidity (N=3760)** |
| Disability |  |  |  |  |  |
| Difficulties in IADLs | 7% (5%, 9%) | 37% (35%, 40%) | 26% (24%, 28%) | 16% (14%, 18%) | 39% (36% , 41%) |
| Difficulties in ADLs | 3% (2%, 5%) | 35% (32%, 37%) | 24% (22%, 26%) | 14% (12%, 17%) | 36% (33%, 39%) |
| Work productivity |  |  |  |  |  |
| Early retirement | 12% (8%, 17%) | 20% (16%, 24%) | 20% (17%, 24%) | 19% (15%, 24%) | 21% (17%, 26%) |
| Number of days of sick leave at main job | 5.05 (2.48, 7.62) | 21.89 (18.14, 25.63) | 16.05 (13.56, 18.54) | 10.08 (7.89, 12.27) | 23.06 (19.02, 27.11) |
| Social participation |  |  |  |  |  |
| Social participation | 69% (52%, 82%) | 46% (41%, 51%) | 53% (50%, 56%) | 58% (55%, 61%) | 46% (41%, 51%) |
| Note: All estimates adjusted with sample weight. Disability was estimated among full sample. Early retirement and Sick leave days were estimated among working-age population (aged under 60).Social participation was estimated among current non-working population.  Data are proportion or estimated mean (95% CI) | | | | | |
