# Supplementary material for: The association between mental-physical multimorbidity and disability, work productivity, and social participation in China: a panel data analysis
Source: BMC Public Health. 2021 Feb 18;21:376. doi: 10.1186/s12889-021-10414-7 (PMC7890601; doi:10.1186/s12889-021-10414-7)
Supplement: Supplementary file 4 — Additional file 4. Association between multimorbidity and disability, work productivity and social participation, models including interactions between physical conditions and depression. Table A3 presents the results from the model that included interactions between number of physical chronic conditions and depression. [file 12889_2021_10414_MOESM4_ESM.docx]

Additional File 4

Title: Association between multimorbidity and disability, work productivity and social participation, models including interactions between physical conditions and depression

| A Table 3 Association between multimorbidity and disability, work productivity and social participation, models including interactions between physical conditions and depression | | | | | |
| --- | --- | --- | --- | --- | --- |
|  | Difficulties in IADLs (n=11116) AOR (95% CI) | Difficulties in ADLs (n=11162) AOR (95% CI) | Early retirement (n=5261) AOR (95% CI) | Number of days of sick leave  (n=4141) mean (95% CI) | Social participation (n=3301) AOR (95% CI) |
| Number of physical NCDs | **1.28 (1.21, 1.35)** | **1.5 (1.41, 1.59)** | **1.31 (1.17, 1.45)** | **1.26 (1.13, 1.40)** | 1.08 (1.00, 1.16) |
| Depression | **3.68 (2.94, 4.61)** | **5.61 (4.36, 7.22)** | 0.97 (0.63, 1.50) | **2.21 (1.49, 3.28)** | **0.63 (0.43, 0.92)** |
| Physcial chronic conditions * Depression ^a^ | 0.99 (0.92, 1.06) | **0.87 (0.80, 0.93)** | 1.11 (0.95, 1.28) | 0.99 (0.85, 1.16) | 0.97 (0.87, 1.08) |
| Age group (ref: 45-54) |  |  |  |  |  |
| age 55-64 | **1.43 (1.21, 1.69)** | **1.66 (1.37, 2.00)** | **1.97 (1.55, 2.51)** | 0.92 (0.72, 1.17) | 0.91 (0.68, 1.23) |
| age 65-74 | **2.21 (1.82, 2.67)** | **2.47 (2.00, 3.05)** |  |  | 0.93 (0.68, 1.29) |
| age 75+ | **4.07 (3.13, 5.29)** | **3.78 (2.84, 5.04)** |  |  | **0.66 (0.44, 0.97)** |
| Female gender | **1.46 (1.27, 1.67)** | **1.18 (1.02, 1.37)** | **4.59 (3.38, 6.24)** | 0.89 (0.70, 1.13) | 1.06 (0.85, 1.32) |
| Married | 1.03 (0.86, 1.23) | 0.97 (0.79, 1.17) | 0.98 (0.60, 1.60) | 1.37 (0.85, 2.19) | **0.74 (0.57, 0.95)** |
| Agricultural hukou | 1.09 (0.91, 1.32) | 1.18 (0.97, 1.44) | **0.33 (0.23, 0.46)** | 1.33 (0.92, 1.92) | **0.48 (0.37, 0.62)** |
| Rural residency | **1.41 (1.21, 1.63)** | **1.23 (1.05, 1.44)** | **0.24 (0.18, 0.33)** | 1.25 (0.97, 1.63) | 1.19 (0.95, 1.50) |
| Region (ref:east China) |  |  |  |  |  |
| Middle China | **1.2 (1.02, 1.42)** | **1.87 (1.56, 2.25)** | 0.82 (0.59, 1.13) | 1.11 (0.84, 1.48) | 0.94 (0.73, 1.20) |
| West China | 1.12 (0.95, 1.32) | 1.14 (0.95, 1.37) | **0.58 (0.42, 0.81)** | 1.17 (0.89, 1.55) | **0.66 (0.51, 0.86)** |
| Northeast China | **1.78 (1.37, 2.31)** | **1.72 (1.29, 2.31)** | 1.11 (0.66, 1.86) | 1.35 (0.82, 2.24) | 0.76 (0.53, 1.10) |
| Family size (ref: 1-2 members) | |  |  |  |  |
| 3-4 members | 1.07 (0.93, 1.23) | 1.06 (0.91, 1.22) | 1.02 (0.77, 1.33) | 0.9 (0.69, 1.17) | 0.95 (0.77, 1.18) |
| 4+ members | **1.22 (1.04, 1.44)** | 0.94 (0.79, 1.13) | **1.52 (1.09, 2.11)** | 1.06 (0.78, 1.46) | 1.08 (0.83, 1.40) |
| Education level (ref:illiterate) |  |  |  |  |  |
| Primary | **0.6 (0.51, 0.71)** | **0.75 (0.63, 0.89)** | 0.83 (0.58, 1.19) | 0.79 (0.58, 1.07) | 1.27 (0.98, 1.64) |
| Secondary | **0.48 (0.39, 0.58)** | **0.61 (0.49, 0.75)** | 1.2 (0.86, 1.69) | 0.79 (0.58, 1.08) | **1.4 (1.04, 1.87)** |
| Tertiary | **0.38 (0.28, 0.52)** | **0.43 (0.31, 0.60)** | 0.87 (0.56, 1.35) | 0.76 (0.52, 1.12) | **2.8 (1.84, 4.27)** |
| HH consumption per capita (ref:Q1) | |  |  |  |  |
| Q2 | 1.04 (0.89, 1.22) | 0.99 (0.83, 1.17) | 0.85 (0.60, 1.19) | 1.13 (0.83, 1.54) | 1.18 (0.90, 1.54) |
| Q3 | 1.06 (0.90, 1.25) | 0.89 (0.74, 1.06) | 1.1 (0.79, 1.53) | 1.03 (0.76, 1.40) | 1.09 (0.84, 1.42) |
| Q4 (richest) | 1.11 (0.94, 1.32) | 1 (0.83, 1.20) | **1.68 (1.21, 2.34)** | **1.49 (1.07, 2.07)** | 1.3 (0.99, 1.70) |
| Work type (ref:farming) |  |  |  |  |  |
| Formally Employed |  |  |  | **0.32 (0.23, 0.44)** |  |
| Self-employed |  |  |  | **0.65 (0.43, 0.99)** |  |
| Family business |  |  |  | 1.3 (0.63, 2.70) |  |
| 2015 | **1.21 (1.08, 1.35)** | **1.46 (1.29, 1.65)** | **1.59 (1.28, 1.97)** | 1.07 (0.85, 1.35) | 0.98 (0.82, 1.17) |
| Note: The regression model is adjusted for all socio-demographic covariates. AOR, Adjusted odds ratio; CI, confidence interval.Bond font indicate significance at 5% level. | | | | | |
| Generalized linear model with gamma distribution and log link function is used to estimate the association between multimorbidity and the number of days of sick leave at main job. Random-effect logistic models are used for other outcomes. | | | | | |
| a: Physcial chronic conditions * Depression indicates the coefficient or AOR for the interaction term between number of physcial chronic conditions and depression. | | | | | |
