# Supplementary material for: The association between mental-physical multimorbidity and disability, work productivity, and social participation in China: a panel data analysis
Source: BMC Public Health. 2021 Feb 18;21:376. doi: 10.1186/s12889-021-10414-7 (PMC7890601; doi:10.1186/s12889-021-10414-7)
Supplement: Supplementary file 5 — Additional file 5. Sensitivity analyses on the association between multimorbidity and productivity loss among different age groups. Table A4 presents the results of the sensitivity analyses on the association between multimorbidity and productivity loss using different samples. We repeated the analysis using a sample of respondents aged under 55 years, which is the mandatory retirement age for female white-collar workers. In addition, we used a cut-off at 65 years, given the possibilities that people may continue working after the mandatory retirement age. [file 12889_2021_10414_MOESM5_ESM.docx]

Additional File 5 (pdf)

Title: Sensitivity analyses on the association between multimorbidity and productivity loss among different age groups

| Table A4 Sensitivity analyses on the association between multimorbidity and productivity loss | | | | |
| --- | --- | --- | --- | --- |
|  | **Sample of respondents aged < 55 years** | | **Sample of respondents aged < 65 years** | |
|  | Early retirement (n=3040) AOR (95% CI) | Number of days of sick leave  (n=2472) mean (95% CI) | Early retirement (n=7626) AOR (95% CI) | Number of days of sick leave  (n=5735) mean (95% CI) |
| Number of physical NCDs | **1.41 (1.26, 1.59)** | **1.22 (1.11, 1.36)** | **1.35 (1.27, 1.44)** | **1.29 (1.21, 1.37)** |
| Depression | 1.17 (0.83, 1.64) | **2.7 (1.98, 3.69)** | 1.09 (0.90, 1.32) | **2.09 (1.72, 2.55)** |
| Age group (ref: 45-54) |  |  |  |  |
| age 55-64 |  |  | **2.53 (2.04, 3.13)** | 0.91 (0.74, 1.11) |
| age 65-74 |  |  |  |  |
| age 75+ |  |  |  |  |
| Female gender | **6.09 (3.88, 9.56)** | **0.71 (0.52, 0.98)** | **3.91 (3.09, 4.95)** | 0.93 (0.76, 1.13) |
| Married | 1.73 (0.75, 3.97) | 1.36 (0.67, 2.73) | **0.55 (0.39, 0.78)** | 1.15 (0.79, 1.67) |
| Agricultural hukou | **0.42 (0.26, 0.65)** | 1.49 (0.90, 2.47) | **0.31 (0.24, 0.40)** | 1.03 (0.76, 1.40) |
| Rural residency | **0.24 (0.16, 0.36)** | 0.99 (0.70, 1.39) | **0.27 (0.21, 0.34)** | **1.44 (1.16, 1.79)** |
| Region (ref:east China) |  |  |  |  |
| Middle China | 0.75 (0.49, 1.16) | 1.18 (0.81, 1.70) | 0.88 (0.68, 1.13) | 0.96 (0.76, 1.22) |
| West China | **0.52 (0.33, 0.80)** | 1.16 (0.81, 1.66) | **0.6 (0.46, 0.78)** | 1.21 (0.96, 1.53) |
| Northeast China | 0.74 (0.34, 1.60) | 0.61 (0.30, 1.23) | 1.44 (0.97, 2.15) | 0.97 (0.65, 1.47) |
| Family size (ref: 1-2 members) | |  |  |  |
| 3-4 members | 1.23 (0.83, 1.82) | 0.94 (0.65, 1.36) | 0.98 (0.79, 1.20) | 0.89 (0.72, 1.10) |
| 4+ members | 1.38 (0.86, 2.22) | 1.16 (0.75, 1.78) | 1.28 (0.99, 1.64) | 0.94 (0.73, 1.21) |
| Education level (ref:illiterate) | |  |  |  |
| Primary | 0.72 (0.44, 1.17) | 0.77 (0.51, 1.15) | 1.1 (0.84, 1.44) | 0.89 (0.69, 1.13) |
| Secondary | 0.94 (0.61, 1.46) | 0.85 (0.56, 1.27) | **1.49 (1.13, 1.96)** | 0.88 (0.67, 1.14) |
| Tertiary | 0.82 (0.46, 1.45) | 0.85 (0.51, 1.41) | 1.02 (0.70, 1.49) | 0.82 (0.58, 1.16) |
| HH consumption per capita (ref:Q1) | |  |  |  |
| Q2 | 0.78 (0.48, 1.26) | 1.51 (0.98, 2.33) | 0.95 (0.73, 1.23) | 1.12 (0.87, 1.45) |
| Q3 | 1.06 (0.67, 1.68) | 1.23 (0.81, 1.86) | 1.13 (0.87, 1.46) | 0.98 (0.76, 1.27) |
| Q4 (richest) | 1.54 (0.98, 2.42) | **1.71 (1.12, 2.62)** | **1.8 (1.39, 2.33)** | **1.45 (1.10, 1.90)** |
| Work type (ref:farming) |  |  |  |  |
| Formally Employed |  | **0.27 (0.18, 0.42)** |  | **0.28 (0.21, 0.37)** |
| Self-employed |  | 0.66 (0.38, 1.14) |  | **0.68 (0.47, 0.97)** |
| Family business |  | 1.26 (0.55, 2.91) |  | 1.16 (0.64, 2.09) |
| 2015 | **1.8 (1.32, 2.43)** | 1.02 (0.76, 1.38) | **1.59 (1.34, 1.87)** | 1.02 (0.85-1.24) |
| Note: The regression model is adjusted for all socio-demographic covariates. AOR, Adjusted odds ratio; CI, confidence interval.Bond font indicate significance at 5% level. | | | | |
| Generalized linear model with gamma distribution and log link function is used to estimate the association between multimorbidity and the number of days of sick leave at main job. Random-effect logistic models are used for other outcomes. | | | | |
